# Overcoming *Burkholderia cepacia* by successful eradication with clinical improvement in cystic fibrosis

**DOI:** 10.5339/qmj.2024.qitc.7

**Published:** 2024-03-25

**Authors:** Mustafa A. Al-Tikrity, Merlin Thomas, Mona Al Langawi, Reem Hasan Mustafa El Ajez

**Affiliations:** 1 Hamad General Hospital, Hamad Medical Corporation, Doha, Qatar Email: maltikrity@hamad.qa; 2 Department of Clinical Medicine, Weill Cornell Medicine, Doha, Qatar; 3 Clinical Pharmacy, Department of Pharmacy, Hamad General Hospital, Doha, Qatar

**Keywords:** Cystic fibrosis, *Burkholderia cepacian*, Eradication therapy, Clinical improvement

## Background

Cystic fibrosis (CF) is characterized by viscous airway mucus that provides a fertile media for opportunistic pathogens. Chronic colonization with *Burkholderia cepacia* (*B. cepacia*) is associated with an increased risk of respiratory failure and mortality.^1^ Eradication therapies are reported in the literature. However, a standardized protocol has not yet been established.^2^

## Case Presentation

A 28-year-old female with homozygous delta F508 mutation of CF colonized with methicillin-sensitive Staphylococcus aureus (MSSA) and Pseudomonas aeruginosa (PA) suffered from multiple CF-related diseases, including diabetes, liver, and pancreatic insufficiency. She was admitted to hospital with abdominal pain and constipation for 3 days and was diagnosed with intestinal obstruction. She was treated with ileostomy and PEG tube feeding. During her hospitalization, she reported increased daily productive cough with green phlegm and no other symptoms. There was a decrease in lung function in percentage of predicted forced expiratory volume in one second (ppFEV1) from 65% (1.66 liters) to 55% (1.39 liters), along with a first isolate of the sensitive strain of *B. cepacia* in sputum. In view of symptomatic significant decline in lung function, eradication treatment was prescribed according to Gruzelle et al.^2^ (Table 1). She showed significant symptomatic improvement following the induction period with a return of pulmonary symptoms to baseline. Furthermore, ppFEV1 was 58% (1.47 liters) at the end of the consolidation period and 61% (1.56 liters) at 6 months. There were no reported adverse effects or side effects of treatment. Repeat sputum cultures at 3 and 6 months following the initiation of therapy were negative for the growth of *B. cepacia*.

**Table 1.** Eradication protocol for *Burkholderia cepacia*.^2^

	**Medication**	**Dosing**	**Frequency**	**Route**
Induction period (21 days)	Amikacin	30 mg/kg	Daily	IV
	Meropenem	2 g	Every 8 h	IV
	Trimethoprim/Sulfamethoxazole	800/160 mg	Twice daily	Oral
	Azithromycin	250 mg	Daily	Oral
Consolidation period (2 months)	Trimethoprim/Sulfamethoxazole	800/160 mg	Twice daily	Oral
	Amikacin inhaled	500 mg	Twice daily	Nebulized
	Azithromycin	250 mg	Daily	Oral

## Conclusion

We highlighted the successful eradication of *B. cepacia* in a CF patient with the reported regimen. The notable clinical improvement through improvement in lung function and subsequent failure of bacterial growth for 6 months highlights the effectiveness of this regimen in eradicating the initial isolation of *B. cepacia*.

## Conflict of Interest

The authors declare no conflict of interest regarding this case report.
